# Supplementary material for: Dynamic expression and differential requirement of the myocyte fusogen Myomixer during distinct myogenic episodes in the zebrafish
Source: Biol Open. 2025 Dec 18;14(12):bio062305. doi: 10.1242/bio.062305 (PMC12755067; doi:10.1242/bio.062305)
Supplement: Supplementary information [file biolopen-14-062305-s1.pdf]

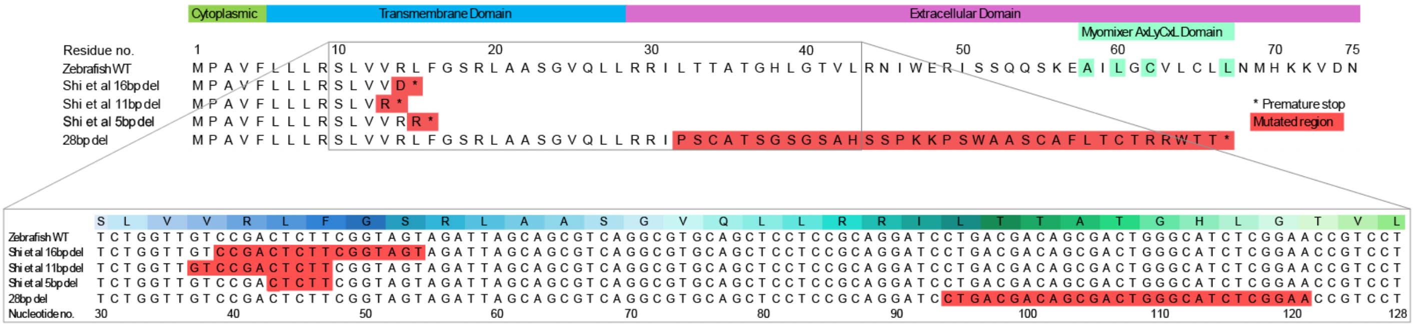

Fig. S1. Protein and nucleotide comparisons of zebrafish *mymx* mutants. Schematic showing the *mymx* protein and coding sequences, comparing the predicted effects of previously published knockout mutations, alongside the 28bp deletion mutant generated and used in this study, to the structure of the WT protein. Protein sequence schematic highlights the cytoplasmic, transmembrane and extracellular regions of Mymx, additionally highlighting the characteristic AxLyCxL domain required for fusion. Mutated regions of the protein are highlighted in red, with premature stops denoted by an asterisk. Cutout box contains the subsequent nucleotide deletions in sequence, highlighted in red, and their comparative locations the protein sequence.

A

		wild type	*mymx*$^{-/-}$
S9 – S12	30 hpf	38 ± 1	104 ± 4
	48 hpf	45 ± 2	117 ± 5
	120 hpf	53 ± 4	126 ± 8
S13 – S16	30 hpf	32 ± 2	87 ± 3
	48 hpf	43 ± 2	99 ± 4
	120 hpf	48 ± 3	109 ± 6
S17 – S20	30 hpf	30 ± 2	67 ± 3
	48 hpf	36 ± 1	66 ± 3
	120 hpf	42 ± 4	83 ± 6

B

		wild type	*mymx*$^{-/-}$
S9 S12	30 hpf	102 ± 3	105 ± 5
	48 hpf	116 ± 4	119 ± 8
	120 hpf	133 ± 10	132 ± 11
S13 – S16	30 hpf	81 ± 5	87 ± 2
	48 hpf	97 ± 2	99 ± 5
	120 hpf	114 ± 7	112 ± 9
S17 – S20	30 hpf	64 ± 3	67 ± 4
	48 hpf	76 ± 2	66 ± 3
	120 hpf	86 ± 6	96 ± 8

Fig. S2. Quantification of cell and nuclear number in different genotypes. Comparison of cell number (A) and number of nuclei (B) per myotome segment at different developmental positions (denoted by somite stage) and time post fertilisation (n=3 embryos per condition). In (A), differences between wild type and *mymx*$^{-/-}$ are significant to $p<0.001$ (www.estimationstats.com). In (B), only S17-S20 at 48 hpf is significant ($p = 0.02$), though this is likely an experimental error.

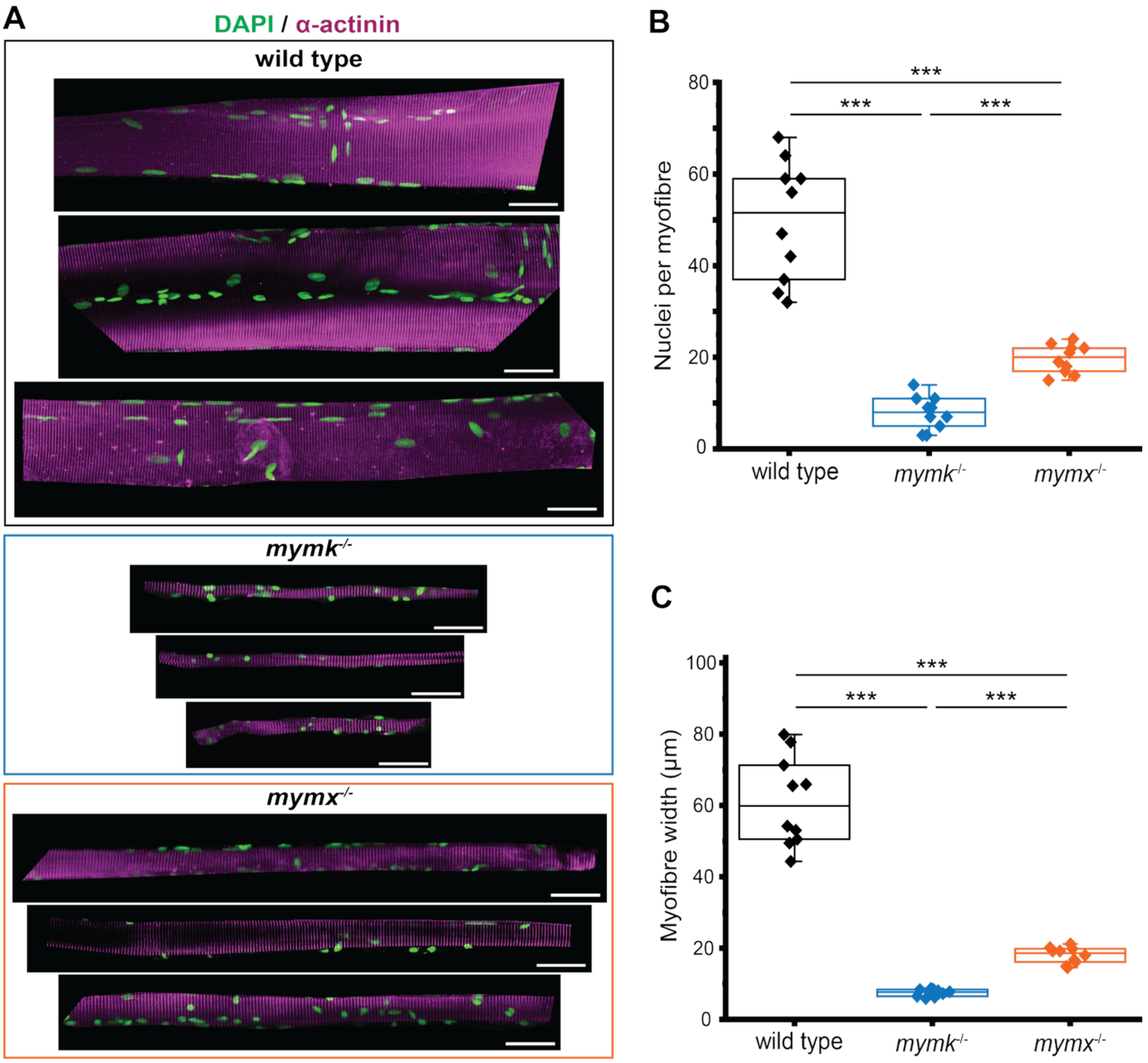

Fig. S3. Skeletal muscle structure of adult zebrafish. (A) Single fast-twitch skeletal muscle fibres isolated from the trunk musculature of 3-month-old wild type, mymk-/- and mymx-/- fish, immunostained for α-actinin (magenta) and DAPI (green). Scale bars = 30 µm. (B) Width of myofibres and (C) number of nuclei per myofibre (n = 10 myofibres from 3 fish per genotype). The difference between groups was calculated using estimationstats.com, with the wild type as the control group: *** $p < 10^{-3}$.

Biology Open • Supplementary information

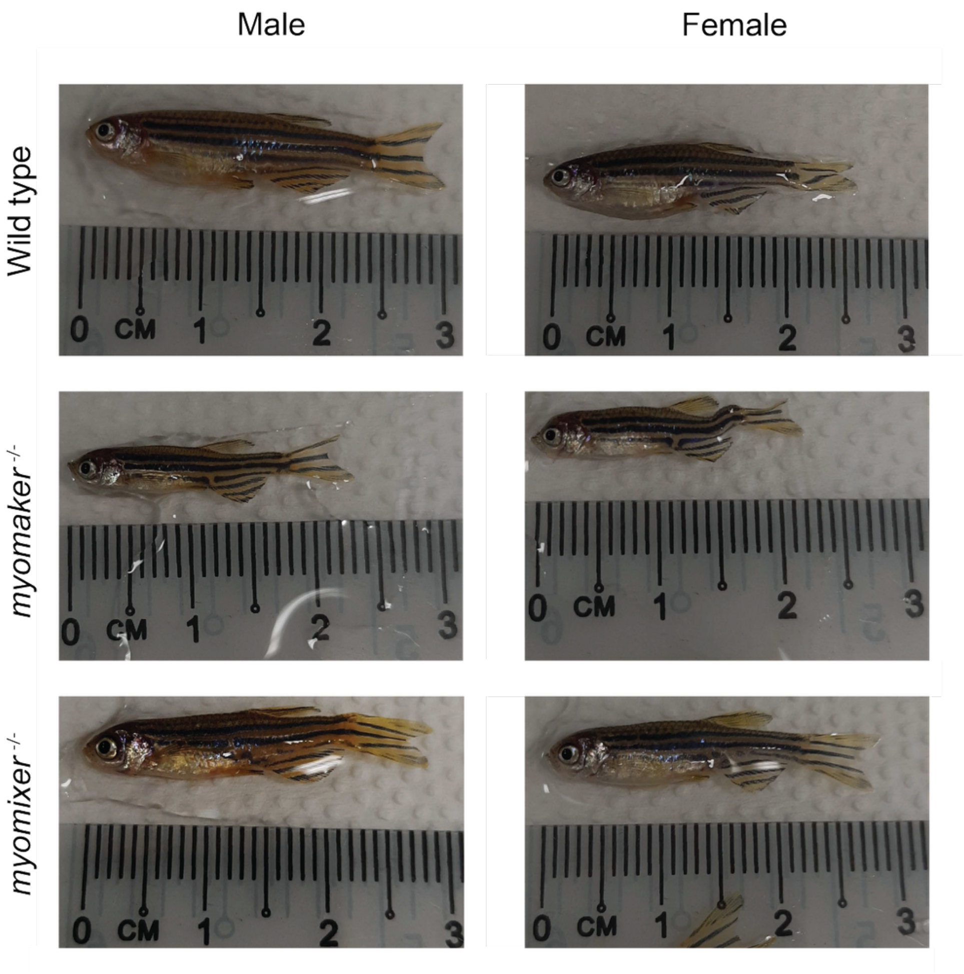

Fig. S4. Mutations in *mymk* and *mymx* impact adult morphology. Representative images of wild type, *mymk*$^{-/-}$ and *mymx*$^{-/-}$ three month-old adult zebrafish.

Biology Open • Supplementary information
